# Supplementary figures and images for: The relationship between distal trunk morphology and object grasping in the African savannah elephant (Loxodonta africana)
Source: PeerJ. 2022 Mar 28;10:e13108. doi: 10.7717/peerj.13108 (PMC8969868; doi:10.7717/peerj.13108)

Distal shapes trajectories for the apple

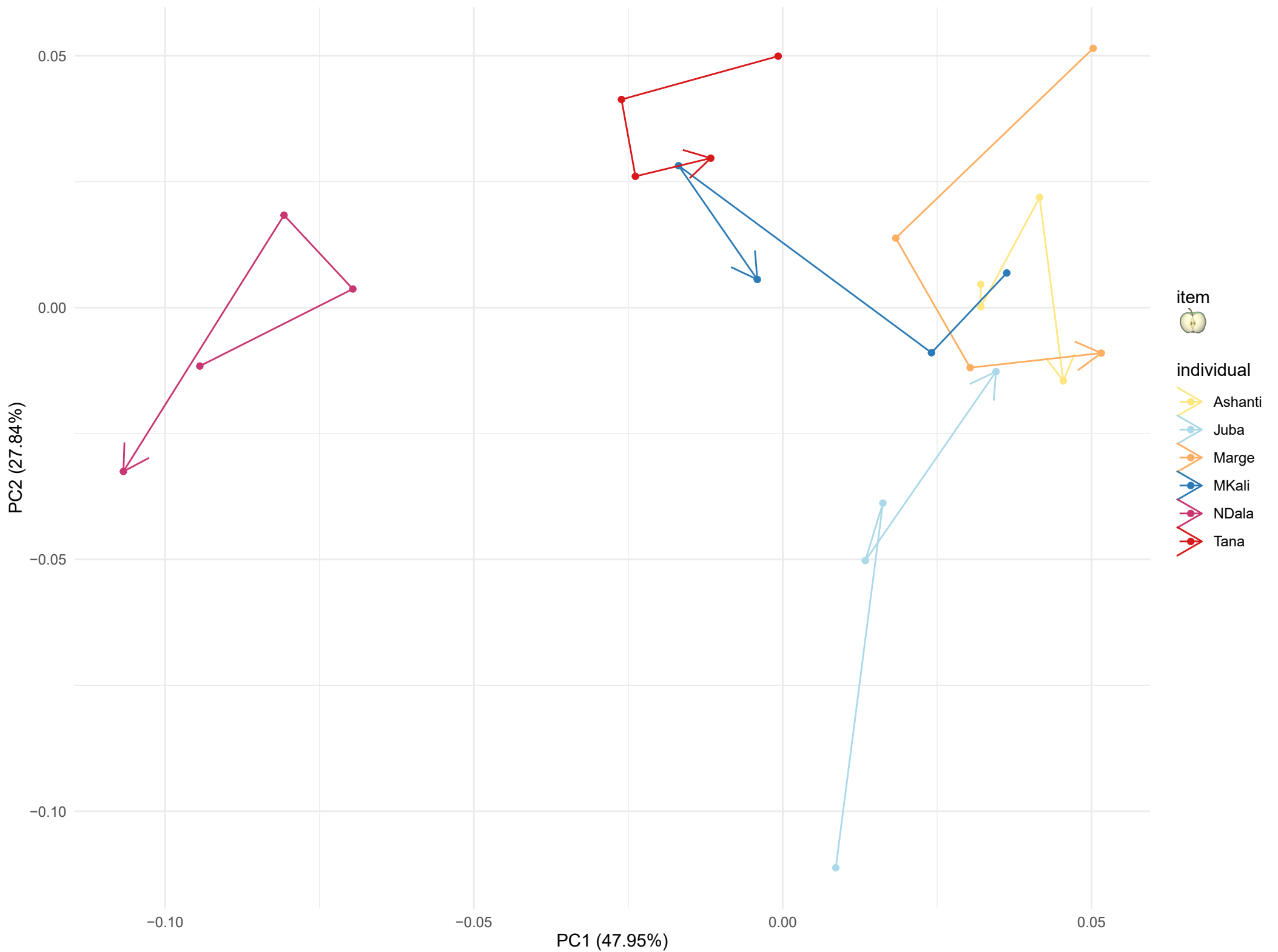

Supplement: Supplemental Information 2 — Supplementary figure linked with Figure 7. [file peerj-10-13108-s002.pdf]

Distal shapes trajectories for the carrot

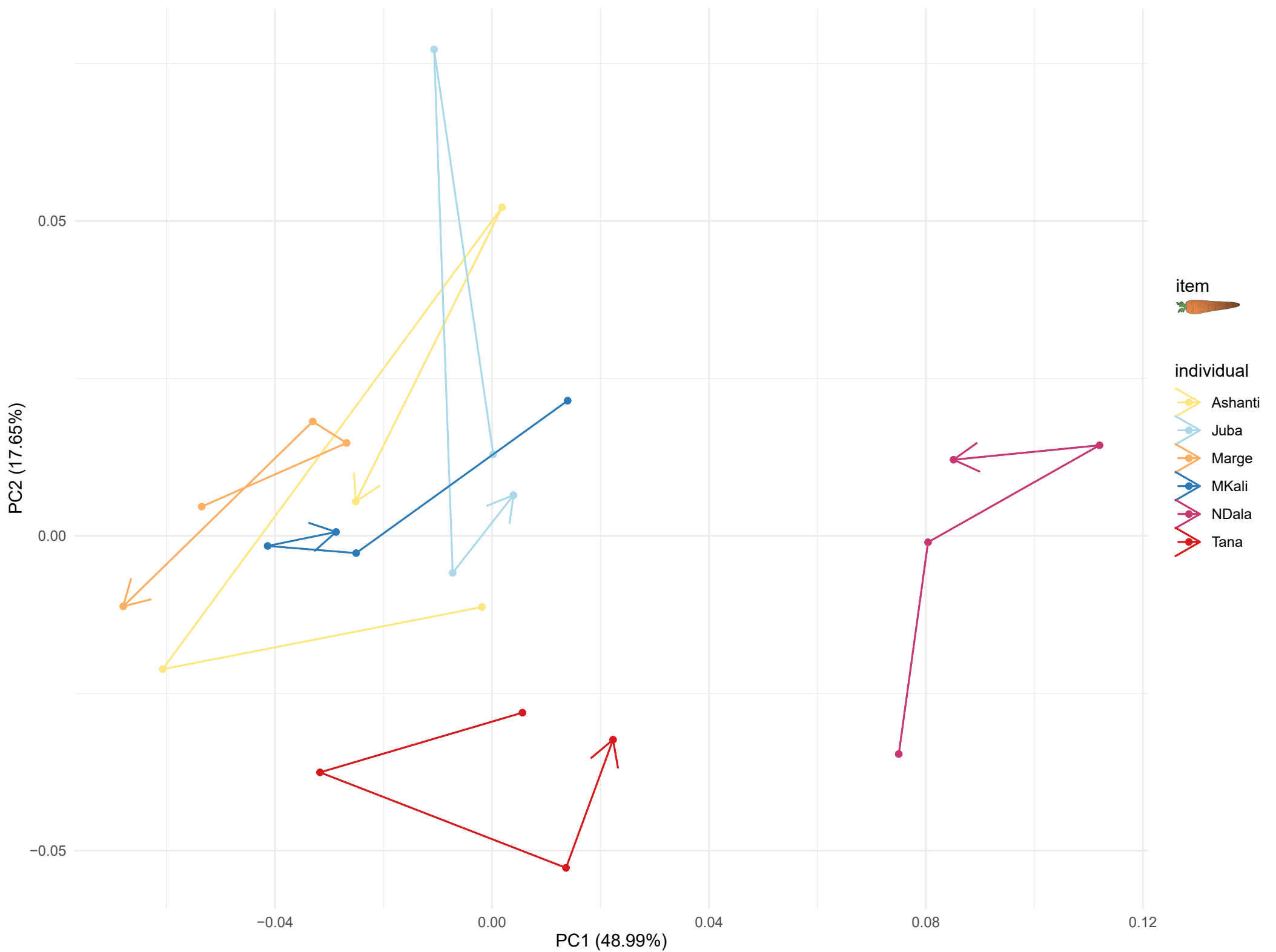

Supplement: Supplemental Information 3 — Supplementary figure linked with Figure 7. [file peerj-10-13108-s003.pdf]

Distal shapes trajectories for the large cube

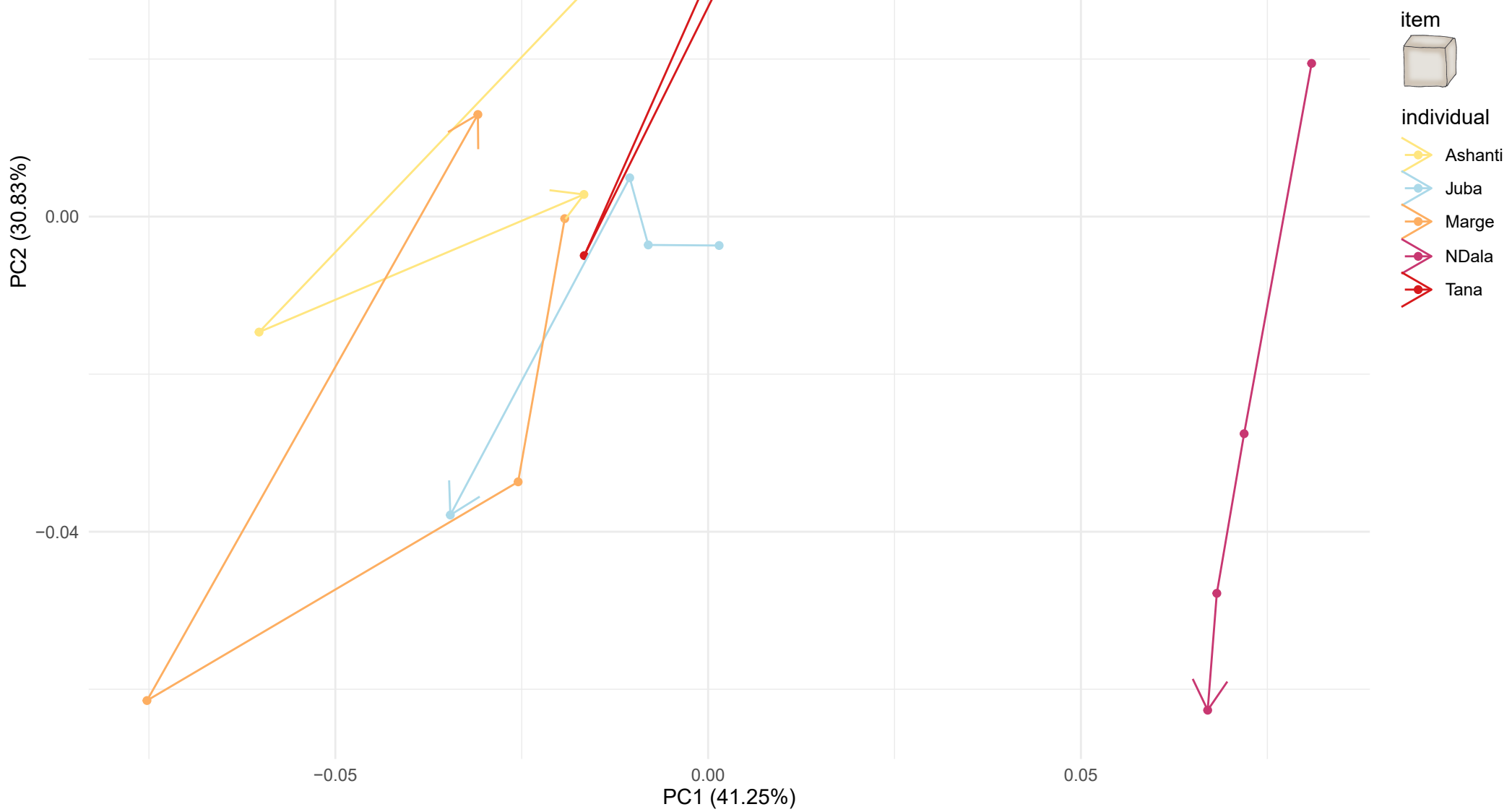

Supplement: Supplemental Information 4 — Supplementary figure linked with Figure 7. [file peerj-10-13108-s004.pdf]

Distal shapes trajectories for the small cube

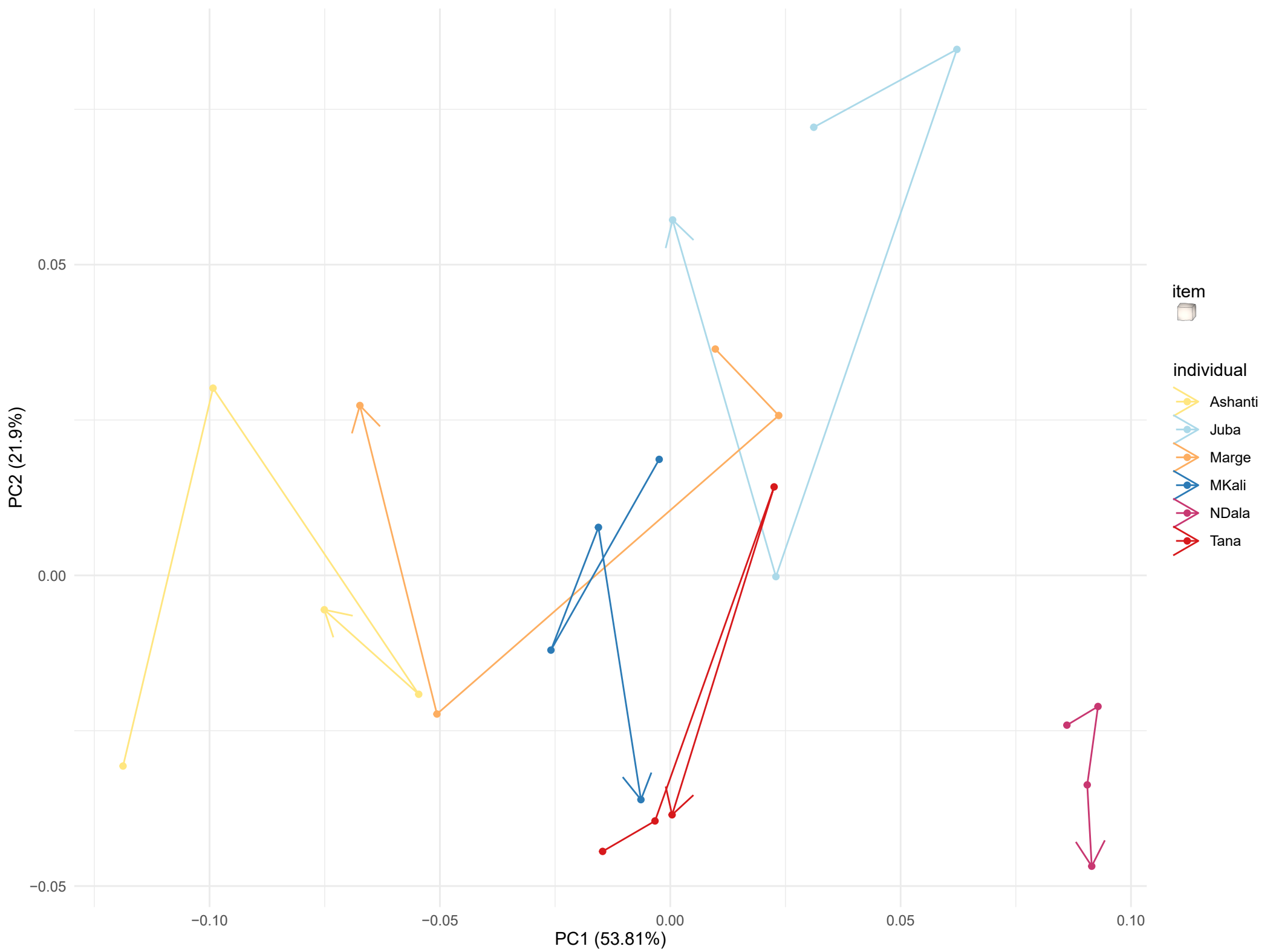

Supplement: Supplemental Information 5 — Supplementary figure linked with Figure 7. [file peerj-10-13108-s005.pdf]
